# Biomass Straw-Derived Porous Carbon Synthesized for Supercapacitor by Ball Milling

**DOI:** 10.3390/ma15030924

**Published:** 2022-01-25

**Authors:** Bixia Jiang, Lin Cao, Qinghua Yuan, Zhuwen Ma, Zhenrui Huang, Zhidan Lin, Peng Zhang

**Affiliations:** 1 Institute of Advanced Wear & Corrosion Resistant and Functional Materials, Jinan University, Guangzhou 510632, China; jiang0903@stu2020.jnu.edu.cn; 2 Key Laboratory of Crop Genetic Improvement of Guangdong Province/Guangdong Provincial Engineering & Technology Research Center for Tobacco Breeding and Comprehensive Utilization, Crops Research Institute, Guangdong Academy of Agricultural Sciences, Guangzhou 510640, China; qinghuay@foxmail.com (Q.Y.); conghuama@163.com (Z.M.); fjsi@163.com (Z.H.)

**Keywords:** porous carbon materials, supercapacitor, tobacco straws, biochar

## Abstract

A large amount of biomass straw waste is generated every year in the world, which can cause serious environmental pollution and resource waste if disposed of improperly. At present, biomass-derived porous carbon materials prepared from biomass waste as a carbon source have garnered attention due to their renewability, huge reserves, low cost, and environmental benevolence. In this work, high-performance carbon materials were prepared via a one-step carbonization-activation method and ball milling, with waste tobacco straw as precursor and nano-ZnO as template and activator. The specific surface area and porous structure of biomass-derived carbon could be controlled by carbonization temperature, which is closely related to the electrochemical performances of the carbon material. It was found that, when the carbonization temperature was 800 °C, the biochar possesses maximum specific surface area (1293.2 m^2^·g^−1^) and exhibits high capacitance of 220.7 F·g^−1^, at 1 A·g^−1^ current density in a three-electrode configuration with 6 M KOH aqueous solution. The capacitance retention maintained about 94.83% at 5 A·g^−1^ after 3000 cycles. This work proves the porous biochar derived from tobacco straws has a great potential prospect in the field of supercapacitors.

## 1. Introduction

With the surge of population, the booming of agriculture has led to a rapid increase of agricultural waste. China, as the largest agricultural country in the world, produces a large amount of agricultural and forestry biomass waste every year, and most of the biomass waste is mainly sourced from crop straw, forest straw and organic waste [1,2,3]. According to the China Statistical Yearbook 2019, China had produced about 8.65 × 10^9^ tons of straw in 2019 [4], which occupied a large part of the total biomass resources in China, and this proportion will continue to rise in the future. However, only a small amount of straw waste was being recycled, used as feed and fertilizer materials [5,6]. Most of the straw was still disposed of in the traditional way: burning or direct disposal. The decay of straw waste would absorb a large amount of nitrogen, phosphorus and potassium and lead to a decrease in soil fertility, while open burning would also produce a large amount of carbon dioxide and other pollutants, leading to serious environmental pollution, and causing waste of renewable resources due to its renewability [7,8,9]. Therefore, promoting the comprehensive utilization of straw resources and transforming into high added value materials are of great practical significance for environmental protection, resource conservation and economic development.

In recent years, the productions of biomass carbon materials from agricultural wastes have become a research hotspot in energy storage. Biomass is an ideal precursor for preparing various types of carbon materials because of its abundant reserves, convenient transportation and sustainable development [10,11]. There have been many reports on the utility of biological waste to prepare biochar, such as banana peels [12], corn cobs [13], baobab husks [14], pomegranate peels [15], and wheat straw [16] and so on. These biochar materials are widely used in various fields because of their low cost, wide sources, considerable economic value and diversified morphology, such as soil improvers [17], wastewater treatment adsorbents [18], catalysts [19], etc. Specifically, the biochar as electrode materials for supercapacitors would be a promising application, which suffers from the competitive relationship between the abundant electrochemically active sites and the fast ion transfer channel from biochar. However, it still faces tremendous challenges in simultaneously designing and preparing biomass-derived carbons with excellent capacitive and rate performance.

Regarding to the high performance of supercapacitors, ideal carbon materials should own high specific surface area, hierarchical porous structure (combination of mesoporous and microspores), and heteroatom doping [20,21,22]. The physicochemical properties of biomass-derived carbon materials play a key role in the electrochemical performance of supercapacitors, which all depend on the preparation process of high-performance biomass carbon materials [23]. Therefore, it is important to develop an efficient and simple method to prepare biochar electrode materials with porous structures. Currently, chemical activation, such as KOH, ZnCl_2_, or H_3_PO_4_, is commonly used to optimize the porous structure of carbon. Sun [24] prepared activated carbon by hydrothermal processing and chemical activation using hemp stems as the carbon source and KOH as the activator, with a specific surface area of 2879 m^2^·g^−1^ and specific capacitance of 160 F·g^−1^. Wu [25] prepared bioactive carbon with KOH and HNO_3_ activation by using almond shell as raw material, with specific surface area of 1363 and 327.7 m^2^·g^−1^ and specific capacitance (1 A·g^−1^) of 272.3 and 286.1 F·g^−1^, respectively. However, despite that the activation would help to produce a high specific surface area of biochar materials during the preparation process, the over-corrosive effect of these activators would produce larger pores, which is harmful for ion transport. In addition, these activators are usually corrosive to instruments and produce acidic or alkaline effluents [26,27,28]. Accordingly, many new methods have been developed to design the pore structure of carbon materials, among variety methods; template carbonization could control the morphology and pores structure of carbon materials efficiently [29,30,31,32]. Jian [33] prepared a mesoporous carbon material with nano-ZnO as a template using egg white as carbon source, with specific capacitance of 205 F·g^−1^ (current density of 0.5 A·g^−1^) and excellent cycling performance (97% retention after 3000 cycles). Xu [34] prepared a bio-porous carbon material by using rhombus shell as carbon precursor, nano-ZnO as template and KOH as activator, with a high electrochemical property with specific surface area up to 1537 m^2^·g^−1^ and specific capacitance of 128 F·g^−1^ (5 mV·s^−1^).

Nano-ZnO has been used as a template material because of several unique characteristics. On the one hand, it has been shown that nano-ZnO could be used both as a hard template to produce mesoporous and also as an activator to prepare hierarchical porous carbon; therefore, nano-ZnO is a promising dual-role additive to prepare porous carbon [35,36]. On the other hand, ZnO could be easily removed by using acids or alkaline for its essentially amphoteric properties.

The aim of this work is to explore a simple and efficient carbonization-activation method to prepare straw-derived carbon and develop the economic value of waste tobacco straws. In this study, tobacco straw-derived carbon was prepared by one-step carbonization activation method with ball milling method, with nano-ZnO as the template and activator, and tobacco straws as carbon precursor. Furthermore, the carbonization temperature has a significant influence on the morphology, specific surface area and electrochemical performance. Therefore, the structural properties of the carbon materials obtained at different carbonization temperature were carried out in this paper. This study can provide a new way to prepare electrode materials by carbonization of biomass.

## 2. Experimental Section

### 2.1. Materials

Tobacco straws were collected from Crops Research Institute, Guangdong Academy of Agricultural Sciences (Guangzhou, China), which were first cleaned with deionized water and absolute ethanol and then dried at 105 °C overnight. The dried straws were crushed into powders and sieved by a 200-mesh. Nano-ZnO (diameter: 30 ± 10 nm) was provided by Shanghai Aladdin Biochemical Technology Co., Ltd. (Shanghai, China).

### 2.2. Synthesis of Porous Carbon Materials

Tobacco straw-derived carbon materials were prepared by using the dual effects of nano-ZnO with one-step carbonization and activation method. Typically, tobacco straw powder and nano-ZnO were mixed in weight ratio of 1:1 by ball milling (QM-3SP2, Nanjing, China) for 2 h at 350 rpm [35]. Then the mixture was transferred into nickel crucible and placed in tubular furnace for carbonization at 700–900 °C (5 °C·min^−1^) for 2 h under N_2_ atmosphere. The products were washed with excess 1 M HCl and deionized water, then dried in the vacuum at 80 °C overnight. According to the different carbonization temperature of tobacco straw powder and nano-ZnO mixture, the obtained carbon samples were named as TCZn-X, where X represents the carbonization temperature. Based on our previous research [37], for comparison, the carbon sample prepared from tobacco straw at 800 °C (5 °C·min^−1^) under N_2_ for 2 h without nano-ZnO was noted as TC-800.

### 2.3. Materials Characterization

The morphologies and microstructures of the carbon materials were observed by field emission scanning electron microscope (FESEM, Zeiss, ULTRATM 55, Jena, Germany) and field-emission transmission (TEM, JEOL, JEM-2100F, Tokyo, Japan). The crystallographic phases and composition analysis were measured using X-ray diffraction (XRD, Rigaku Ultima IV instrument, Tokyo, Japan), and Raman spectra were recorded by Raman spectrometer at wavelength of 633 nm (LR-3, Varian, Palo Alto, CA, USA).

Furthermore, the pore size distributions and specific surface areas of the carbon materials were measured by a BSD-PS2 (Beijing, China). The surface area porosity analyzer calculated via the Brunauer–Emmett–Teller (BET) method. The nitrogen-adsorption/desorption was performed on the carbon samples at 77.3 K after they had been degassed at 573 K for 3 h.

### 2.4. Electrochemical Characterization

All the electrochemical properties of the carbon samples were carried out on a CHI 760E workstation (CH Instruments, Austin, TX, USA) by a typical three-electrode configuration in a 6 M KOH solution at room temperature. A platinum sheet (10 mm × 10 mm) and a saturated calomel electrode were used as counter electrode and reference electrode (SCE), respectively. For the preparation of working electrodes, sample powders, polytetrafluorethylene (PTFE) and acetylene black were thoroughly mixed at a mass ratio of 8:1:1, and then coated onto the nickel foam (10 mm × 10 mm) and dried at 80 °C overnight. Afterwards, the working electrodes were pressed at a pressure of 10 MPa and used for further studies.

The electrochemical test in this experiment mainly includes cyclic voltammetry (CV), galvanostatic charge-discharge (GCD), and electrochemical impedance spectroscopy (EIS). For the three-electrode configuration, the specific capacitances were calculated from galvanostatic charge-discharge curve as follows:

(1) $$C_{s}=\frac{I\Delta t}{m\Delta V}$$

where *C_s_* (F·g^−1^) is the specific capacitances of the working electrode, *I* (A) is the current, Δ*t* is the discharge time, *m* (g) is the weight of the active materials on the working electrode, Δ*V* (V) is the range of the potential [38].

## 3. Results and Discussion

### 3.1. Materials Characterization

Based on our previous work [37], compared with carbon materials directly prepared at different pyrolysis temperatures, the biochar (TC-800) obtained directly by pyrolysis at 800 °C had the better performance, so TC-800 was used as the control group.

**Figure 1:**
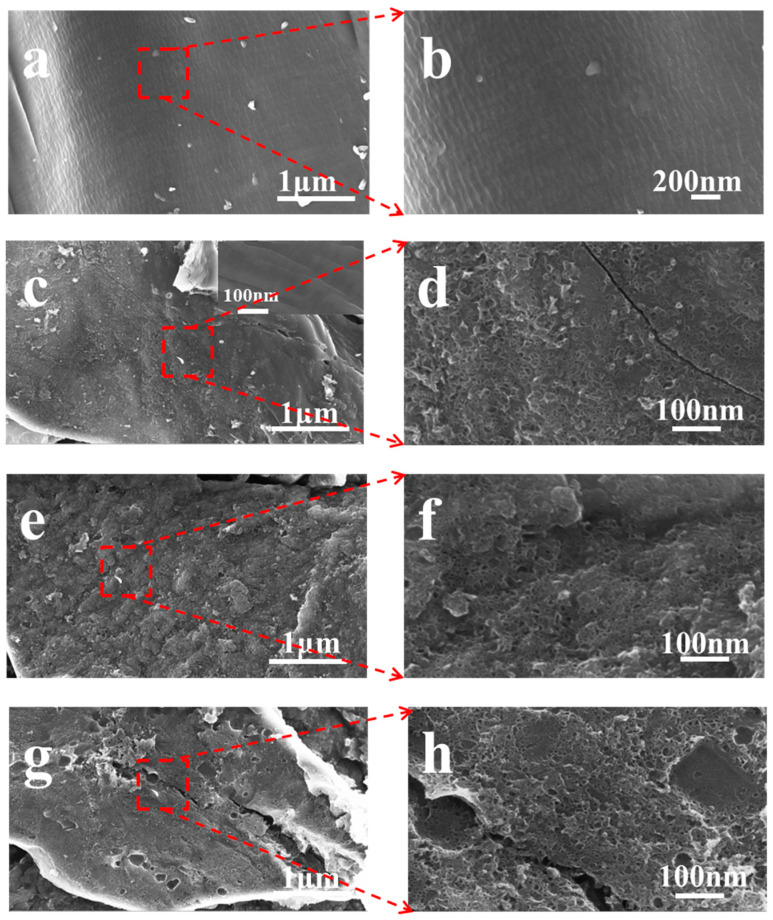
SEM images of (**a**,**b**) TC-800; (**c**,**d**) TCZn-700; (**e**,**f**) TCZn-800; (**g**,**h**) TCZn-900.

**Figure 2:**
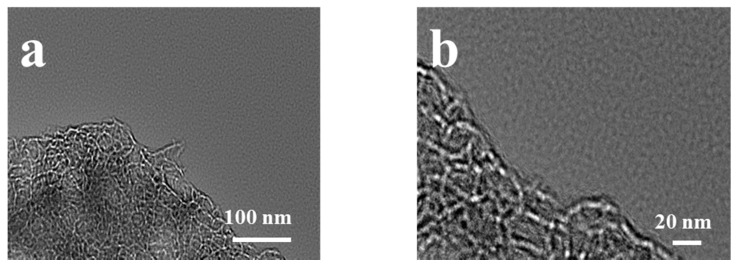
TEM images of TCZn-800 sample at (**a**) 100 nm and (**b**) 20 nm.

The surface morphologies and porous properties of TC-800, TCZn-700, TCZn-800 and TCZn-900 were analyzed based on SEM (Figure 1) and TEM (Figure 2). As shown in Figure 1a,b, the surface of TC-800 was relatively smooth and covered by large-size lumps, while the sample was directly pyrolyzed at 800 °C without nano-ZnO. In contrast, when the nano-ZnO was introduced into the tobacco carbon sample, the surface of the TCZn-X samples showed a much rougher surface and looser structure with abundant interconnected pores as shown in Figure 1c–h. Meanwhile, the temperature was a key factor to affect the surface morphology of TCZn-X samples. The convex part of TCZn-700′s edge is smooth with almost no pores, while the concave part is rough with few vesicles (Figure 1c,d), which may be due to insufficient carbonization temperature and incomplete carbonization. As shown in Figure 1e,f, there are a larger number of pores of smaller diameters and various depths of TCZn-800, which makes the roughness of TCZn-800 larger than that of TCZn-700. This is consistent with its TEM images (Figure 2a,b), and the high-resolution TEM further shows the abundant microspores and mesoporous of the carbon skeleton of TCZn-800. The surface roughness of TCZn-900 is similar to that of TCZn-800. However, the pore size of TCZn-900 is larger and looser, and the pore distribution is uneven. These results may be attributed to the high carbonization temperature led to excessive carbonization, pore collapse and deformation. The S_BET_ and S_micro_ of the samples at different temperatures are different, mainly due to the weak electronic conductivity and small specific surface area of the samples annealed at low temperature (TCZn-700); the samples annealed at high temperature (TCZn-900) have a high degree of graphitization and crystal Due to the high degree of density, some pores are blocked, and the specific surface area is limited. The porous structure of the TCZn-X samples could be attributed to the activation and template effect of nano-ZnO [30]. In the preparation of carbon materials, nano-ZnO was used as hard template and activating agent. The site-occupying effect of nano-ZnO was similar to silica, mainly produced mesoporous. Also, nano-ZnO can etch some carbon atoms at high temperature to create micropores, as well as enlarge the meso/macropores according to the following reaction [36]:

ZnO + C = Zn + CO

The porous structure was found to be essential for high surface area in electrode material, which could facilitate constructing a better electrolyte diffusion and ion storage container for supercapacitors [39].

**Figure 3:**
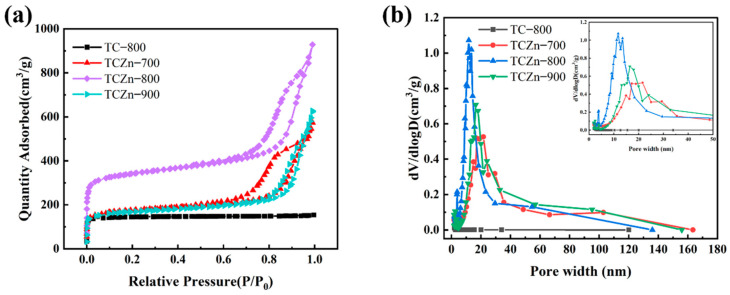
(**a**) N_2_ adsorption/desorption isotherms and (**b**) pore size distributions for all samples.

The specific surface area and porous structure of the samples were calculated by N_2_ adsorption/desorption isotherms (Figure 3a,b). Obviously, all the samples exhibit typically combined type Ⅰ and Ⅳ isotherms. The Ⅳ-type hysteresis loops as observed in isotherms of all the TCZn-X samples, suggesting the existence of abundant mesopores [40]. The above results are consistent with TEM analysis. Moreover, the specific areas of the samples were calculated by Brunauer–Emmett–Teller (BET) method and Barret–Joyner–Halenda (BJH) model. As shown in Table 1, the addition of nano-ZnO did have a significant effect on the specific surface area (SSA), the SSA of TC-800 without nano-ZnO is only 569.4 m^2^·g^−1^, while the SSA is effectively increased with nano-ZnO as template and activator, especially the SSA of TCZn-800 is high to 1293.2 m^2^·g^−1^, which was beneficial to improve the SSA of carbon materials as supercapacitor electrodes. These results could be attributed to the dual role of nano-ZnO during the activation process [41]. The mesoporous hierarchical structure in the TCZn-X samples contributed to enhancing both storage and transport of electrolyte ions.

**Figure 4:**
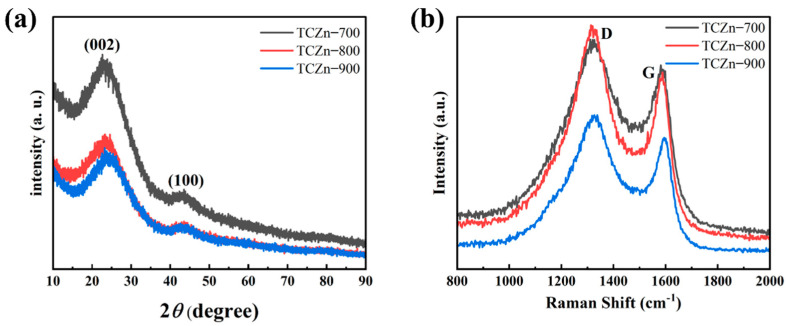
(**a**) XRD patterns and (**b**) Raman spectra of TCZn-700, TCZn-800 and TCZn-900.

The crystalline structures of all the TCZn-X samples were further analyzed using XRD and Raman spectroscopy. As shown in Figure 4a, all the samples show a similar XRD pattern, and there are two broad diffraction peaks centered at 26° and 44°, just like most other reported biomass-derived charcoal [42,43]. These peaks correspond to the (002) and (110) planes of graphite, suggesting the amorphous carbon [44]. The Raman spectra (Figure 4b) of all the TCZn-X samples display two characteristic peaks; one is D peak at the 1335 cm^−1^, and the other is G peak at the 1590 cm^−1^, which are typical peaks of the carbon materials. The D and G peaks are related to disordered carbon structure and graphitic lattice, respectively. In general, the intensity ratio of the D and G band (I_D_/I_G_) is used to indicate the degree of graphitization [45,46]. The I_D_/I_G_ values of TCZn-700, TCZn-800 and TCZn-900 are 1.12, 1.31, and 1.24, respectively. Significantly, the I_D_/I_G_ value of TCZn-800 is the highest of all the samples. Meanwhile, the higher the I_D_/I_G_ value is, the higher the graphitization degree of carbon material is, and the higher the conductivity of TCZn-X as electrode material is [47]. Therefore, the graphitization degree and the conductivity of TCZn-800 was all the highest, which is probably due to the proper temperature, promoting the activation and templating effect of nano-ZnO, which would result in a large number of defects.

### 3.2. Electrochemical Characterization

**Figure 5:**
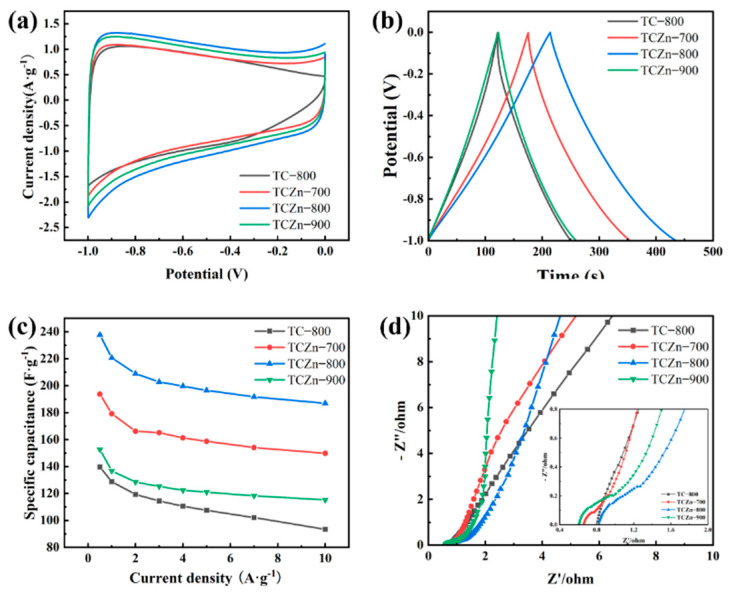
Electrochemical properties of all samples (**a**) CV curves at 10 mV·s^−1^; (**b**) GCD curves at 1 A·g^−1^; (**c**) Specific capacitance at different current densities and (**d**) Nyquist plots.

**Figure 6:**
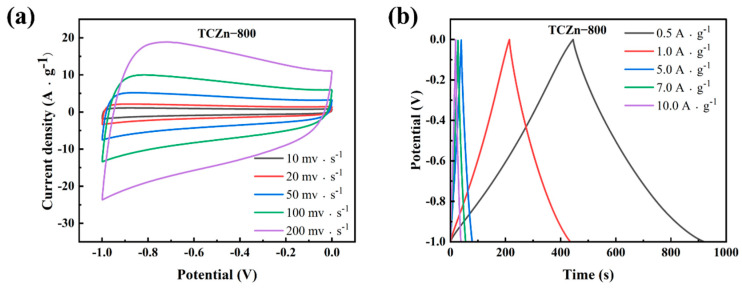
(**a**) CV curves at different scan rates and (**b**) GCD curves at different current densities of TCZn-800.

The electrochemical performances of all the samples were carried out using a three-electrode configureuration in 6 M KOH aqueous solution electrolyte (Figure 5 and Figure 6). The CV curve reflects the ions adsorption of the electrode material, which is related to the charge storage mechanism of EDCL. As shown in Figure 5a, the CV curves of TCZn-700, TCZn-800 and TCZn-900 exhibit rectangular shape at 10 mV·s^−1^ scan rate with a voltage range of −1 to 0 V, indicating their superior double-layer capacitor behavior. Unlike the other three samples, the CV curve of TC-800 showed a distorted rectangular shape, suggesting a poor EDLC performance. This could be attributed to the ion-sieve effect of samples, which was caused by the lack of mesoporous. The area bounded by the CV curve of the TCZn-800 is distinctly larger than others, demonstrating its best specific capacitance, which is consistent with the TEM and BET results. In addition, under different carbonization temperatures, the CV enclosed areas of the TCZn-X are quite different, indicating that their capacitances are different. It can be seen that when the temperature increasing, the area of the CV curve becomes larger, but when the temperature reached 900 °C, the area of the CV curve decreased. This is mainly because the low-temperature annealed sample (TCZn-700) was insufficient carbonization, which resulted to a weak electronic conductivity and small specific surface area. While the sample was annealed at an excessive high-temperature (TCZn-900), excessive carbonization leads to large pore size, limited specific surface area and high crystallinity. Generally, the larger area of the CV curve, the higher of capacitance, and the stronger adsorption capacity of EDCL for ions [48,49]. So, the carbonization temperature is one of the important factors affecting the performance of biochar materials. The GCD curves of all the samples show an isosceles triangle shape at 1 A·g^−1^ current density (Figure 5b). This phenomenon indicated that all the samples own a favorable reversibility of electrochemical and high coulombic efficiency. Notably, all the TCZn-X show a much longer discharge time than TC-800. Specifically, TCZn-800 showed the longest discharge time, which reflects its advantage of high SSA and pore structure for capacitance improvement. The excellent electrochemical performance of the TCZn-800 sample could be attributed to the activation and templating of nano-ZnO and appropriate carbonization-activation temperature [50].

Figure 5c summarized the rate capability of all samples under different densities as derived from their GCD curves. It is further verified that porous structure and high specific surface area are helpful in enhancing the TCZn-X’s specific capacitance. The specific capacitance of TC-800, TCZn-700, TCZn-800 and TCZn-900 were 128.7, 179.2, 220.7 and 136.7 F·g^−1^ at 1 A·g^−1^ current density. But when the current density is up to 10 A·g^−1^, the specific capacitances were 93.4, 149.8, 186.9 and 115.2 F·g^−1^, respectively. Obviously, TCZn-800 sample shows the highest specific capacitance; the specific capacitance retention is 84.69% from 1 A·g^−1^ to 10 A·g^−1^ current density. Figure 6 shows CV curves and GCD curves of TCZn-800 at different scan rates and current densities to further investigate its electrochemical performance. Its CV curve still maintained a quasi-rectangular outline at a high scan rate (Figure 6a); the GCD curves also exhibited typical electrochemical capacitor behavior over a wide range of current densities (Figure 6b), suggesting its excellent capacitive behavior.

The electrochemical impedance spectroscopy (EIS) was carried out over the frequency range from 0.01 Hz to 100 kHz to further explore the electrochemical properties of TCZn-X samples. Figure 5d displays the Nyquist plots of all samples, which can be divided in two distinct parts, the low frequency (R_s_) and the high frequency (R_ct_: charge transfer resistance), respectively [51]. At low frequency, the more vertical the plots slope, the faster the diffusion of ions, which would result in a higher conduction performance [52]. So, the ionic diffusion capacity of all the samples is faster. Moreover, the R_ct_ values of all the samples are fairly low, suggesting good conductivity.

**Figure 7:**
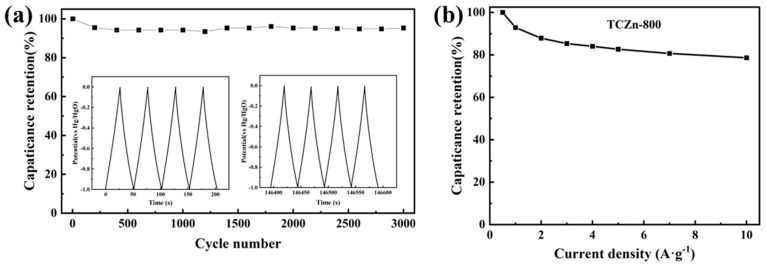
(**a**) 3000-cycle stability test at 5 A·g^−1^ and (**b**) the capacitance retention with different current densities of TCZn-800.

To further check the long cycle life of TCZn-800, it was tested under a current density of 5 A·g^−1^. As shown in Figure 7, the GCD curves of the first few cycles and the last few cycles are almost isosceles triangles, and the capacitance retention is about 94.83% after 3000 cycles. These above results confirm the excellent cycle stability of TCZn-800.

## 4. Conclusions

With tobacco straw as a carbon source and nano-ZnO as a hard template and activator, tobacco straw-derived porous activated carbon was successfully prepared by one-step carbonization activation method and ball milling method. The results show that different carbonization temperatures have a profound effect on the surface morphology of bio-porous carbon materials, and then affect their electrochemical performance. When the carbonization temperature was 800 °C, the biochar (TCZn-800) had a high specific surface area (1293.2 m^2^·g^−1^) and mesoporous structure. TCZn-800 exhibits superior performance with a capacitance of 220.7 F·g^−1^ at 1 A·g^−1^ in a three-electrode configureuration and excellent cycle life (about 94.83% capacitance at 5 A·g^−1^ after 3000 cycles). This work displays a simple method for preparing excellent-performance porous carbon electrode using biomass-straw and provides ideas for the resource utilizations of waste tobacco straws and other biomass resources.

## Figures and Tables

**Table 1:** Structural characteristics of the as-prepared materials.

Samples	*S_BET_* (m^2^·g^−1^)	*S_micro_* (m^2^·g^−1^)	*V_total_* (cm^3^·g^−1^)	*D_ap_* (nm)
TC-800	569.4	557.7	0.23	1.67
TCZn-700	650.4	503.4	0.88	5.41
TCZn-800	1293.2	1057.0	1.43	4.43
TCZn-900	625.4	498.0	0.96	6.17

*S_BET_:* specific surface area; *S_micro_*: micropore surface area; *V_total_*: pore volume; *D_ap_*: average pore diameter.

## Data Availability

The data presented in this study are available on request from the corresponding author.
